# Supplementary material for: Are carbon emissions trading and green financial instruments synergistic? -Comprehensive quantitative research based on content analysis
Source: PLoS One. 2024 Mar 7;19(3):e0298601. doi: 10.1371/journal.pone.0298601 (PMC10919601; doi:10.1371/journal.pone.0298601)
Supplement: S1 Data — (ZIP) [file pone.0298601.s001.zip › Data/地级市Z_F工作报告.zip!/║■▒▒/╢⌡╓▌╩╨/└┤╫╘╣1⁄2╓┌║┼í╛┬φ┐╦╩2╛▌═°í┐_2014.docx]

各位代表：现在，我代表市人民政府向大会作政府工作报告，请予审议，并请各位市政协委员和列席人员提出意见。

　　一、2013年工作回顾2013年，面对复杂多变的国内外经济形势，全市上下深入贯彻党的十八大和习近平总书记视察湖北重要讲话精神，按照“竞进提质、效速兼取”的总要求和市委的决策部署，沉着应对，难中求进，实现了经济社会快速健康发展。

　　———经济保持持续增长，综合实力不断增强。面对经济下行压力，坚持应急与谋远并举，统筹推动各项改革发展措施，保持了经济稳定增长。预计全年完成地区生产总值630.5亿元，增长10.8％，人均GDP达到9400美元；固定资产投资565亿元，增长28%；规模以上工业增加值361亿元，增长13%；地方公共财政预算收入38.4亿元，增长16.4%；全社会消费品零售总额205亿元，增长13%；实际利用外资1.62亿美元，增长16.3%；外贸出口1.74亿美元，增长10.4%；城镇居民人均可支配收入21620元，增长12%；农民人均纯收入10300元，增长13%。主要指标完成预期目标，增幅快于全省平均水平。

　　———转型升级步伐加快，结构调整成效显现。启动实施“重点产业倍增计划”，出台促进工业经济平稳增长、扶持市场主体发展等一系列措施，筹措10.2亿元财政间歇资金，撬动各方资金注入实体经济，推动经济逐步好转。全市工业用电量从8月份开始实现正增长，扭转了连续20个月负增长的局面，重点行业和骨干企业开始走出低谷。全年新增规模以上工业企业49家，新增营业收入过亿元企业45家。规模以上工业实现销售收入1160亿元，增长14.4%。新型建材、高端装备制造、生物医药等新兴产业稳步发展。高新技术产业快速发展，完成增加值89.5亿元，增长16.5%，占GDP的比重达到14.2%。实施省级以上科技项目41项，产学研成果转化项目数是上年的1.65倍。以电子商务为代表的生产性服务业迅猛发展。

　　18平方公里的电子商务基地建设顺利，成功引进了亚马逊、普洛斯、唯品会、当当网、苏宁易购等国内外知名电商企业，葛店开发区获批全省首批电子商务示范基地。金融业健康发展，全市银行增量贷存比达125%，居全省第一，市和三个区齐获“全省金融信用市、区”称号。

　　———招商引资成果丰硕，发展后劲更加充足。抢抓国内外产业转移机遇，出台一系列激励和优惠政策，大员上阵、高位推进招商引资，全市形成了大招商、招大商、抓发展的浓厚氛围。先后引进了南山集团葛店临港新城、东湖高新梧桐湖信息产业基地、南都新能源等一批重大项目落户。全年新签约亿元以上项目105个，到位资金361亿元，同比增长41%。

　　扎实开展“项目建设年”活动，以项目建设夯实发展基础，全市在建亿元以上项目增长38%；实施技术改造项目124个，完成投资42.6亿元，增长37%。总投资74亿元的鄂州电厂三期工程取得重大进展，两台100万千瓦超超临界机组即将动工。顾地总部、波尔亚太三期等52个重点工业项目建成投产。

　　———农业基础地位巩固，城乡一体化质量提升。以农业增效、农民增收为中心，持续加大农业投入，战胜春涝伏旱灾害，稳定了农业生产。粮食总产量35.5万吨，增长2.9%，实现“十连增”；水产品总产量43.9万吨，增长10.8%；全市农业实现增加值71.6亿元，增长12.1%。新建生态农业示范基地56个，注册地理标志商标9件，全国首个有机农产品检测认证中心落户我市，长港峒山新社区被列为全国现代生态农业创新示范基地，我市被命名为武昌鱼标准化养殖示范市和国家“粮安工程”示范市。农业产业化快速推进，新增省级龙头企业4家、农民专业合作社113家、家庭农场210户，农产品加工产值达到210.3亿元，增长31%。城乡一体化加速发展，农村生产生活条件明显改善。投资3.5亿元实施水利工程建设；新建通村公路200公里。投资5.25亿元实施国土整治23.5万亩。认真落实各项强农、惠农、扶农政策，减轻农民负担工作获得省级表彰。

　　顺利开展第三轮整村推进扶贫开发。投入6000万元实施“清洁乡村”工程，在全省率先建立了城乡一体化垃圾收集处理系统。因地制宜推动新社区建设扩点、强面，建设初具规模的农村新社区76个。去年7月，习近平总书记到峒山新社区视察，对我市城乡一体化和新农村建设工作给予了充分肯定。

　　———新型城镇化高位推进，新区建设取得新进展。把新型城镇化作为发展新引擎，初步形成了城乡统筹、全域联动、多点发力的新格局。确立了建设葛店开发区、三江港区、鄂州开发区、鄂城新区、花湖开发区、梁子湖生态文明示范区、红莲湖新区等七大沿江滨湖新区。以主城区为龙头、七个新区为支撑、特色镇为链接、新社区为基础的“四位一体”新型城镇体系构架基本形成。各新区按照一个主导产业、一个建设开发主体、一套高起点的规划、一批高标准的基础设施项目、一个明确的开发建设时序的“五个一”思路，与中省大型投资集团开展战略合作。南山集团与葛店临港新城、中核集团与鄂城新区、鄂旅投与红莲湖新区、中交投与三江港新区签订了战略合作协议。十个特色镇实施基础设施建设项目55个，完成投资1.4亿元。将生态文明理念融入新型城镇化建设，启动了“三边”植树和环梁子湖生态林带建设，我市被列入国家第一批水生态文明建设试点城市，梁子湖区被列为全国生态文明建设试点，500平方公里范围全面退出一般工业。

　　———城市功能逐步完善，市容面貌明显改观。市委、市政府作出了“五城同创”的决策部署，努力创建国家卫生城市、国家环保模范城市、全国旅游标准化试点示范城市、全国文明城市、国家生态园林城市。全市动员，全民参与，城市建设和管理水平明显提升。主城区实施基础设施项目34个，完成投资14.3亿元，投资规模创历史新高。武黄城际铁路建成即将通车。火车站广场、寒溪生态广场、鄂州大道南互通、江碧路改造、背街小巷改造、洋澜湖综合治理、主次干道亮化绿化等重点工程顺利实施。三江港铁路货场、吴楚大道、黄鄂高速以及葛华水厂等工程正加快建设。梧桐湖新区、葛店开发区、鄂州开发区等园区基础设施建设力度加大，承载能力大为增强。以“五城同创”为契机，以创促建、以创促管。城市管理重心下移，引入城市管理公共评价机制，开展治超治限、出店占道经营、户外广告、建筑渣土、停车秩序等多项专项整治，城市面貌明显改善。大力实施“碧水、蓝天、宁静、循环、生态”五大工程，加大环保执法力度，主要污染物减排目标顺利实现。

　　———综合改革不断深化，创新活力竞相迸发。政府、市场、社会三者关系进一步理顺，对58家市直行政单位的18大类行政职权进行了全面清理，缩减市级行政审批事项至74项，市级行政审批项目收费全部取消。政府服务效能进一步提升，电子政务平台建成开通，74项行政审批事项实现“外网受理、内网审批”，发展环境进一步优化。深化农村产权制度改革，启动了第二批指标交易试点，完成指标交易金额2.1亿元。城乡网格化管理制度进一步完善，受理处置范围拓展到城市管理、食品药品安全监管等领域。公共服务进一步下沉，打造“一刻钟”公共服务圈，66项公共服务事项实现了社区现场办理。与此同时，公立医院改革取得了新的突破，公共资源交易管理制度改革、国有资产管理运营制度改革、事业单位分类改革等顺利推进。

　　———民生保障能力提升，社会保持安定和谐。坚持民生优先，公共财政向民生领域倾斜。全年各类民生支出33.2亿元，占公共财政支出比重达72%。认真办理“十件实事”，有效解决了一批与群众生活密切相关的实际问题。全力推进创业就业工作，发放小额财政贴息贷款1.1亿元，新登记各类市场主体1.1万户，新增小微企业618家，城镇新增就业2万人。五大社会保险新增扩面3万人，开展被征地农民就业和社会保障试点，对全市所有80岁以上老年人发放了老龄津贴，城乡低保水平和“三无”对象供养标准分别提高23.7%和140%。实现了城乡居民大病保险全覆盖，对参保人员实行大病二次报销。探索开展社区养老服务，新建社区互助养老服务中心103家。

　　开工建设各类保障性住房4600套，解决了2500户困难家庭住房问题。发放公积金贷款3.9亿元。妥善解决拆迁安置历史遗留问题，大部分拆迁户入住新居。新增出租车100辆，缓解了城区“打的”难。筹资近2亿元，完成了60所义务教育学校标准化建设，在全省率先完成义务教育均衡发展改革试验任务。基本公共文化服务体系建设取得新进展，市博物馆新馆建成并向市民开放，“周周乐”等群众文化活动日益丰富多彩。在全省率先实现数字农家书屋村级全覆盖。开展吴王城遗址保护工作。城市社区卫生服务机构综合改革基本完成，265所村卫生室实现“四化”达标，提前两年完成省定任务。居民消费价格指数涨幅控制在2.6%，物价保持基本稳定。改革食品药品安全和卫生计划生育管理体制。全年保持了安全生产形势稳定局面，得到国务委员王勇的充分肯定。深入开展矛盾纠纷排查化解工作，严厉打击各类违法犯罪活动，社会保持和谐安定。

　　一年来，政府自身建设不断加强。认真贯彻中央“八项规定”和省委“六条意见”，推行部门预算制度，严控“三公”经费，厉行节约。推动信息公开，坚持依法行政，规范行政行为。强化行政监察和审计监督，加大惩治腐败力度。切实转变机关作风，行政效能明显提高。认真办理人大建议和政协提案370件，办结率和满意率达到100%。自觉接受法律监督、民主监督和社会监督。与此同时，其他各方面的工作都取得了新的成绩。

　　各位代表，回首过去的一年，我们在挑战中抢抓机遇，在困难中砥砺前行，各项工作取得了可喜的成绩。这是省委、省政府和市委正确领导、全市人民奋力拼搏的结果，也是全市多年来打基础管长远、厚积薄发的结果。在此，我代表市人民政府，向全市广大干部群众，向全体人大代表、政协委员和各民主党派、工商联、无党派人士、各人民团体，向驻鄂州解放军指战员、武警官兵，向在鄂州的中省单位和广大外来投资者，向所有关心支持鄂州发展的海内外朋友，表示崇高的敬意和衷心的感谢！在充分肯定成绩的同时，我们也清醒地看到，全市经济社会发展仍然存在不少困难和问题：经济总量不大、发展不够仍然是鄂州最大的实际；产业结构不优、整体抗风险能力不强、发展质量不高的局面仍然没有根本改变；发展不均衡、不协调的问题仍然存在。改革进入攻坚期，重点领域和关键环节的深层次矛盾有待破解。对此，我们一定高度重视，采取更加有力的措施加以化解。

　　二、2014年工作安排今年，是全面贯彻党的十八届三中全会精神的开局之年。党的十八届三中全会作出了全面深化改革若干重大问题的决定，开启了改革发展的新征程。纵观国内外大势，我们面临着前所未有的历史机遇，国家区域经济发展重心内移，将长江经济带作为打造中国经济升级版的支撑带，湖北在中部崛起的战略支点地位更加突出。我市在全省“一元多层次”战略中担负着综合改革示范和城乡一体化试点重任，市委六届八次全会作出了全面推进综合改革的工作部署，将进一步释放发展潜能和改革红利，使我市加快发展、转型发展的动力更加强劲。同时，我们也要充分预计到今年经济工作还面临着国内外市场不确定、不稳定的挑战，面临着经济增长“换挡期”、结构调整“阵痛期”和前期刺激政策“消化期”三期叠加的挑战，我们一定要增强忧患意识和危机意识，进一步解放思想，以求真务实、改革创新、只争朝夕的精神和奋发有为的工作状态，凝神聚力，埋头苦干，努力开创鄂州科学发展、跨越式发展新局面。

　　今年政府工作的总体要求是：全面贯彻党的十八大、十八届三中全会、省委十届四次全会和市委六届八次全会精神，坚持“稳中求进、改革创新”的总基调，按照“竞进提质、升级增效”的总要求，把改革创新贯穿于经济社会发展的各个领域各个环节，着力激发市场活力，着力调整经济结构，着力推进沿江滨湖新区开放开发，着力扩大对外开放，着力保障和改善民生，着力加强生态文明建设，努力实现经济持续健康发展和社会和谐稳定，在湖北“建成支点、走在前列”的进程中作贡献、打头阵。

　　今年经济社会发展主要预期目标是：全市地区生产总值增长11%，规模以上工业增加值增长13%，固定资产投资增长22%，地方公共财政预算收入增长12%，社会消费品零售总额增长13%，招商引资增长20%，城镇居民人均可支配收入增长10%，农民人均纯收入增长11%，城镇登记失业率控制在4.5%以内，完成省下达的节能减排任务。

　　围绕以上目标，重点抓好以下八个方面的工作。

　　（一）大力解放思想，全面深化改革，激活发展内生动力。贯彻落实《全面深化改革行动纲要》，进一步解放思想，进一步提高改革的系统性、整体性、协同性，在重点领域取得新突破。一是全面激发市场活力。建立公平开放透明的市场规则，推行负面清单管理模式，实行统一的市场准入制度。以“先照后证”为突破口，推进工商注册便利化，放开公司注册资本最低限额。改革市场监管体系，落实“预许可”制度，进一步向市场放权，启动全市统一的社会征信体系平台建设。二是创新新型城镇化体制机制。创新沿江滨湖新区管理体制，推进“一级政府三级管理”，全面理顺市、新区、新社区职责事权，探索新区与行政区融合、政府管理与社会管理结合的路径。深化农村产权制度改革，加快推进“五权两指标”交易。完善具有鄂州特色的农村新社区建设模式，推进户籍制度改革，促进农民就地市民化。争取开展全国中小城市综合改革试点。三是深化行政审批制度改革。进一步简政放权，最大限度减少政府对微观事务的管理，最大限度减少审批事项，优化政务运行机制，扩大“网上政务”覆盖范围，建成综合性公共服务电子政务平台。推进中介机构与主管部门彻底脱钩。推进事业单位分类改革、公共资源交易管理制度改革和国有资产管理运营制度改革。四是推进社会事业改革创新。深化教育、就业、医疗卫生、公共财政体制、社会保障和救助服务等社会事业改革，实现发展成果更多更公平惠及全体人民。建立和完善公共服务社会化体系，激发社会组织活力，支持服务类社会组织发展。积极稳妥推进金融、教育、文化、医疗等服务业领域有序开放。五是建立系统完善的生态文明制度。开展自然资源确权登记，建立自然资源资产产权制度和用途管制制度。按照主体功能分区划定生态红线。

　　建立生态文明目标指标体系和考核办法。落实最严格的水资源保护制度，全面建立生态环境责任追究制度，用制度保护生态环境。

　　（二）坚持产城一体，加快新区开放开发，构建新型城镇化多点支撑格局。加快沿江滨湖7个新区开放开发，拓展城市发展空间，引导产业和人口向新区集聚，实现产城融合发展。一是高标准编制新区规划。按照每个新区的发展定位，高标准编制7个新区规划，按照“产城融合”理念，开展新区总体规划、产业规划、控制性详规以及土地规划修编等工作，引领7个新区既错位互补，又独立成篇。强化规划的刚性约束，加强规划的协同性、整体性、系统性和前瞻性，统一布局产业平台、基础设施、公共事业，坚持一张蓝图干到底。二是大力建设基础设施。根据新区规划和开发时序，合理确定年度工作计划和投资目标，大手笔、大力度推进新区基础设施建设。总结借鉴葛店开发区拆迁工作和梧桐湖新区安置被征地农民的经验，创新模式，加快拆迁、供地、项目落户等服务工作。集中连片推进新区道路、水电气、通讯、污水处理等设施建设，拉开新区主干路网骨架，增强新区承载力。三是强化新区产业支撑。坚持科学发展一步到位，主动承接国内外产业转移，按照各新区自然禀赋、产业特色，实行差异化产业发展定位，出台引导政策，推动项目向新区集中，招商引资项目原则上放在新区，促进新区快速发展。

　　（三）实施创新驱动，加快转方式调结构，打造鄂州经济升级版。依靠市场主体，强化创新驱动，加快构建结构优化、技术先进、竞争力强的现代产业体系。一是培育发展新兴产业，加快形成新集群。加强葛店开发区与东湖高新区产业对接，发展壮大生物医药、新能源、新材料、高端装备制造、电子信息等高新产业集群。建立完善以企业为主体、产学研相结合的技术创新体系，推动创新成果产业化。

　　加快湖北科技企业加速器、梧桐湖创意产业园等创新平台建设，引进和培育一大批创新型企业落户孵化成长。力争高新技术产业增加值增长16%，占全市GDP比重达14.5%。二是改造提升传统产业，努力再造新优势。推进信息化与工业化融合，运用高新技术和先进适用技术对冶金、建材、机械等传统产业进行升级改造，全面提高企业生产装备科技含量，提升清洁生产、节约生产、安全生产和信息化水平，促进传统优势产业高端化。加强财政资金引领作用，支持企业技术改造、提质扩规，使一批本土企业做大规模、做强品牌，形成优势。三是突破性发展生产性服务业，大力培育新增量。加快发展现代物流、物联网、智慧产业、信息消费等生产性服务业，葛店电商基地今年完成3平方公里配套基础设施建设，争取再引进一批国内外知名电商企业总部和采购、运营、支付及数据中心落户，确保国家级“电子商务示范基地”获批。推进葛店中部物联网产业基地和智慧城市产业园建设。将三江港打造为粮食、食品等大宗商品集散地和货物周转枢纽港。引导金融市场规范发展，吸引外地金融机构到我市落户，鼓励民营资本投向金融行业，发展村镇银行。积极发展总部经济。开展第三次经济普查。实施中小商贸企业成长工程，力争全年新增限上企业40家。大力发展生态旅游业，力争全市旅游总收入突破50亿元。

　　（四）强化工业支撑，实施重点产业倍增计划，做大经济发展“底盘”。牢固树立工业强市的理念，突出抓好工业经济这个龙头，着力夯实基础、培育体系、提升素质，不断做大经济总量。一是围绕市场主体，扶优培“强”。坚持一手抓现有产能的有效发挥，一手抓企业技术改造。支持和培育一批市场前景好、管理水平高、成长性强的民营企业。围绕现有支柱产业实施产业链“建链、补链、强链”战略，形成一批百亿级产业集群。研究差异化扶持措施，鼓励企业通过产能整合、资产整合和上下游合作等手段扩大规模，增强市场竞争力。建立和完善中小企业服务中心。支持有条件的企业在主板和创业板上市，开展融资租赁、股权融资、发债等形式募集发展资金，力争今年新增上市公司1家、新增规模以上企业50家。

　　深入实施品牌强市战略，加快培育一批精品名牌，积极创建全国质量强市示范市。二是着眼开放引进，招大引“强”。招商的劲头只能增不能减，坚持大员上阵抓招商，实行招商奖励政策，掀起新一轮招商引资热潮。围绕重点产业倍增计划，把工业项目和生产性服务业项目放在招商引资的第一位，注重投资强度、税收贡献、科技含量、资源节约、环境友好、集约用地等标准，实行招商选资，力争引进一批投资过亿元、十亿元乃至百亿元的大项目。三是建设重点项目，增量求“强”。继续开展“项目建设年”活动，实施“三个两百”工程，对鄂州电厂三期等重点项目采取专班专责服务制度，切实解决项目落地、建设、投产过程中的各种困难问题，为项目建设搞好全程保姆式服务。切实抓住各种示范试点机遇，精心策划申报一批重大项目，力争有更多的项目进入国家和省里的“笼子”。

　　（五）依靠制度创新，增添农村发展活力，推进城乡一体化。将“三农”工作作为重中之重，以农业强、农民富、农村美为总目标，千方百计促进农业增产、农民增收和农村发展。一是构建生态农业发展体系。整合涉农资金，设立1亿元生态农业发展资金，鼓励金融机构向农村延伸，支持梁子湖生态示范区、百里长港、蒲团至庙岭等生态农业示范带建设。创建国家级水产种业示范场。建立鄂州生态农业投入品生态标准、生产控制标准、质量检验检疫标准，打造一批全国有影响的有机农产品品牌。启动国家有机产品检测认证中心项目建设。二是构建新型农业经营体系。加强农田水利建设，严守耕地红线，确保粮食增产。加大土地流转力度，提高土地规模经营水平。引导和培育农业科技、气象减灾防灾、病虫害防治、市场营销等各类农民专业合作社和新型农业服务组织，力争新增农民专业合作社50家，全市70%以上的农户进入合作服务体系。加强农产品电子商务交易平台建设，力争50%以上农产品加工企业入驻。三是构建农产品加工转化体系。用工业理念谋划农业，加快实施“四个一批”工程，提高农产品加工转化水平。加快省级华容农产品加工园建设，引进培育壮大一批有竞争力的农产品加工龙头企业，推进潮商集团冷链物流、雨润食品等项目落户，力争农产品加工业产值完成240亿元，与农业总产值比重达到1.8:1。四是加强农村新社区标准化建设。把农村新社区作为农村城镇化的基础，完善新社区规划布局，因地制宜建设标准化新社区。重点打造沿长港、葛湖线、燕矶杨叶、蒲团庙岭4条新社区示范带，实现强点、连线、扩面。强化农村新社区的产业支撑。进一步推进基本公共服务下沉。五是大力建设“美丽乡村”。注重将传统村落、自然风貌、文化保护与生态旅游发展相结合，彰显地域特色。推进村庄环境整治，完善城乡垃圾清运机制，推进城乡一体污水处理体系建设，力争3年实现城乡污水、废弃物全收集、全处理。扎实抓好新一轮“三万”活动，促进农村经济社会稳定发展。

　　（六）突出品位提升，扎实开展“五城同创”，彰显现代城市特色。以开展“五城同创”为抓手，完善硬件设施，提高管理水平。一是大力推进基础设施建设。重点推进吴楚大道、鄂州大道南互通、三国吴都风光带三期、葛山大道改造、吴都生态公园、凤凰大桥、重载车专用通道等工程建设；推进城东水厂前期工作，建成葛华水厂；加快老城区燃气管网建设。加快交通工程建设，推进主城区客运枢纽站工程，确保黄鄂高速建成通车，推进鄂咸高速公路前期工作，加快城乡客运公交化改造。开展葛山文化体育中心以及市群艺馆、公共图书馆、档案馆的新馆建设前期工作。加强工程质量、造价、投资等监管。建立多元可持续城市建设投融资机制，力争发行第二期城投债，确保全年完成基础设施投资80亿元。二是着力抓好城市管理。完善“统一领导、权责明确、分级管理、以区街为主、以社区为基础”的城市综合管理体系，全面落实责任主体，形成工作合力。培养一批专家型城市管理人才，改进城管执法方式。有针对性地开展城市管理专项整治，加强明堂市场、吴都古肆、古楼街环境整治；新建和改扩建一批集贸市场；稳妥有序启动老城区、棚户区以及城中村改造；加强小区物业、地下停车场规范管理；加强渣土抛洒、施工环境、夜市摊点、户外广告等集中整治；抓好拆违控违工作。三是深入开展全民创建。充分发挥市民的主体作用，调动和激发全社会参与创建活动的热情。推进网格化管理与城市管理的有效结合。

　　实施“百万市民素质提升工程”，开展城市工商网点规范、诚信、文明经营宣传教育，引导广大市民牢固树立文明意识。力争今年建成省级环保模范城市；纳入全国旅游标准化试点示范城市；通过国家卫生城市初检、国家园林城市复检、全国文明城市试检。

　　（七）建设生态文明，引领绿色发展，共建美丽鄂州。树立绿水青山就是金山银山的理念，把鄂州建设成为人与自然和谐相处、经济社会协调发展的绿色家园。一是加强绿色生态体系建设。全面推进水生态文明城市试点建设，严格水资源管理，建立“湖长”制度，落实湖泊保护责任机制。梁子湖区全面退出一般工业。启动湖网生态修复、梁子湖第二入江通道等工程，完成花马湖港、高桥河、长港等中小河流综合整治。加强矿区综合治理和生态修复，加强山体、林地、湿地和野生动植物资源保护，推进退耕还林、城市“增绿”、“三边”植树、环梁子湖生态林带等工程，积极创建林业生态示范区、绿色示范乡村和森林城镇。二是大力发展低碳经济。加快节能环保产业发展和节能环保技术推广应用。依托鄂州经济开发区大力发展节能环保产业，加快“青阳鄂”循环经济示范区建设。积极发展清洁能源、机电装备再制造、新型节能建材、大宗工业废弃物综合利用等低碳循环经济产业链。

　　加快实施LNG、光伏发电、秸秆资源化利用等示范项目。加大节能减排力度，严格执行节能和环境评价制度，实行环保执法“零容忍、全覆盖”，严控高污染、低能效项目，淘汰落后产能。三是狠抓环境综合整治。采取工程减排和管理控排相结合，强化工业污染防治。对鄂钢、电厂、球团厂、世纪新峰等排放量大、群众反映强烈的重点企业加强在线监控，支持其实施脱硝、脱硫、除尘、废水治理以及废渣、余能综合利用等减排工程。加强洋澜湖综合治理，截堵入湖排污口。推进城市雨污分流系统建设。加强农业面源污染治理，开展机动车尾气强制性检测治理，努力为人民群众创造一个优美宜居的环境。

　　（八）加强社会建设，构筑民生保障体系，增进人民福祉。把人民群众切身利益作为政府工作的出发点和落脚点。一是健全促进就业创业体制机制。完善城乡均等的公共就业创业服务体系，实施就业培训、就业援助和创业扶持工程，全面落实税费减免、小额担保贷款、资金补贴等政策，鼓励全民创业，确保全年新增就业1.8万人。二是建立更加公平可持续的社会保障制度。进一步扩大社会保障范围，实现由户籍人口向常住人口覆盖，加快推进社会保障“一卡通”建设。完善城乡一体的社会救助体系，健全社会救助标准与经济社会发展联动机制，适时提高保障标准。推进农村福利院改造升级，提高城乡养老服务水平。

　　加强社会组织工作和社工人才队伍建设。加强保障性住房建设和管理，新建保障性住房6300套，缓解困难群众住房需求。加快项目拆迁安置工程建设，基本解决拆迁安置遗留问题。推进非公经济组织住房公积金制度建设。

　　妥善安置农村留守儿童、妇女、老年人的生活。集中力量，扎实办好向人民承诺的“十件实事”。三是促进社会事业建设。深化教育领域综合改革，坚持教育优先发展。实施学前教育行动计划，每个乡镇建成一所中心幼儿园。

　　加强农村教学点建设，巩固义务教育均衡发展成果。支持鄂州职业大学办出特色，争创一流高职院校。完善文化体育基础设施，建立现代公共文化管理机制，引进社会资本参与公共文化建设。全面深化医药卫生体制和公立医院改革，完善医疗卫生服务体系，加快市中心医院、精神卫生中心、传染病医院和梁子湖区人民医院建设，确保竣工并投入使用。综合治理出生人口性别比，稳定低生育水平。四是加强社会治理，建设平安鄂州。高度重视农产品质量、食品药品质量监督管理体系，加强生产、加工、流通、消费全过程监管。推进放心粮油市场体系建设。稳妥推进重要民生事项的价费改革，保持物价水平基本稳定。进一步加强工矿企业、道路交通、水上安全、消防和应急管理工作，开展隐患大排查、大整治，确保人民群众生命财产安全。加强社会治安综合治理，完善城乡网格化管理，健全社会矛盾纠纷排查调处机制，高度重视人民群众来信来访，着力解决群众反映强烈的突出问题，拓宽社情民意的诉求渠道，构建社区法律援助服务网络，增强人民群众安全感、满意度，建设平安鄂州，促进社会和谐稳定。

　　三、加快政府自身改革和建设各位代表，新的一年，我们将认真贯彻落实党的十八大和十八届三中全会精神，以群众路线教育实践活动为契机，切实转变政府职能，深化行政体制改革，创新行政管理方式，增强政府公信力和执行力，全面建设责任政府、法治政府、服务型政府、廉洁政府。

　　（一）着力转变政府职能。牢固树立“产业第一、企业家老大”理念，转变政府职能，改进工作作风。充分发挥市场在资源配置中的决定性作用和更好地发挥政府作用，进一步减少和下放行政审批事项，放开应该由企业和社会组织自我服务、自我管理的事项。建立健全政府购买服务制度，扩大购买公共服务范围。严厉打击、遏制招投标过程中破坏市场经济秩序的不法行为。全面开展服务企业直通车制度，加强行政监察，曝光查处一批不作为、慢作为、乱作为的反面典型。

　　（二）着力推进依法行政。推进法治政府建设，强化对权力运行的制约监督，规范自由裁量权，把权力关进制度的“笼子”，提高法治思维和依法行政能力。健全行政决策机制和程序，推行重大行政决策草案社会公布制度，完善听证会、网上征询等公众参与决策机制，完善重大行政决策听取人大代表、政协委员意见制度，推进决策科学化、民主化、法治化。

　　依法加强对政府工程、重点领域、资金使用的行政监察和审计监督。自觉接受市人大及其常委会的监督，主动接受市政协的民主监督，办好人大建议和政协提案。认真听取民主党派、工商联、无党派人士和人民团体的意见，主动接受司法、舆论、公众监督，在监督中不断改进和提升政府工作。

　　（三）着力打造阳光政务。进一步深化政务公开，推进行政权力行使依据、过程、结果公开，让权力在阳光下运行。全面实施部门预算，公开“三公”经费、公开预算、公开政府投资项目竣工决算的审计结果，保障人民群众的知情权。完善政府公报、政府网站、新闻发布会、在线访谈、亲民热线等公开方式，探索利用政务微博、微信等新媒体问政于民、问需于民、问计于民。加快便民信息服务平台建设，让群众共享各类便民资源。

　　（四）着力提升政府执行力。严格落实行政问责制，加强行政效能督查，建立健全重大决策部署贯彻执行情况监督检查机制。完善能力席位标准，建立健全科学奖惩机制，提升政府服务能力和执行力。以群众路线教育实践活动为契机，拓展联系服务群众的新途径，办好顺民意、解民忧、惠民生的实事。始终保持夙夜在公、勤勉奋发的工作状态，以求真务实精神抓好各项工作的落实。以政府的“辛苦指数”，换取鄂州的“发展指数”和群众的“幸福指数”。

（五）着力加强廉政建设。认真贯彻中央“八项规定”和省委“六条意见”，严格执行《党政机关厉行节约反对浪费条例》，着力转变作风，降低行政运行成本。坚持反腐倡廉与改革发展力度统一，健全落实党风廉政建设责任制，全面推进惩治和预防腐败体系建设，做到干部清正、政府清廉、政治清明。严肃查处各类违纪违法案件，毫不留情、绝不手软。对腐败问题“零容忍”，铲除腐败毒瘤，以反腐倡廉实际成效取信于民，形成推动经济社会发展的“正能量”。

各位代表，鄂州正站在一个新的历史起点上，改革发展的任务光荣而艰巨。让我们高举中国特色社会主义伟大旗帜，认真贯彻落实党的十八大和十八届三中全会精神，在省委、省政府和市委的坚强领导下，提神增劲，凝心聚力，改革创新，为推进鄂州科学发展、跨越式发展而努力奋斗！
